# Advancing Evidence-Based Health Innovation in Brazil: A Qualitative Program Evaluation of a Binational Educational Initiative to Build Interprofessional Capacity

**DOI:** 10.3390/nursrep16080279

**Published:** 2026-08-11

**Authors:** Kateryna Metersky, Rivia Rangel, Bryan Koivisto, Gilberto Tadeu Reis da Silva, Candida Canicali Primo, Margareth Santos Zanchetta

**Affiliations:** 1 Daphne Cockwell School of Nursing, Toronto Metropolitan University (TMU), 350 Victoria St, Toronto, ON M5B 2K3, Canada; mzanchet@torontomu.ca; 2 School of Nursing, Federal University of Espírito Santo (UFES), Avenida Marechal Campos, 1468, bairro Maruípe, Vitória CEP 29047-100, ES, Brazilcandida.primo@ufes.br (C.C.P.); 3 Department of Chemistry and Biology, Toronto Metropolitan University (TMU), 350 Victoria St, Toronto, ON M5B 2K3, Canada; bryan.koivisto@torontomu.ca; 4 School of Nursing, Federal University of Bahia (UFBA), Rua Basílio da Gama, s/n—Campus Canela, Salvador CEP 40110-040, BA, Brazil; gilberto.tadeu@ufba.br

**Keywords:** health innovation, international partnerships, qualitative study, Canada–Brazil

## Abstract

**Background:** Health innovation education often lacks focus on local realities and practitioner-led approaches. An international Canada-Brazil academic partnership was developed to address this gap within Brazil’s Unified Health System. **Objectives:** This paper examines attendees’ perceptions of a short-term, binational educational intervention and identifies perceived gaps in innovation training within a resource-constrained health system. **Design:** A qualitative, pedagogically focused program evaluation of a short-term binational educational intervention, using participant reflections and post-course survey responses to examine perceived learning, applicability, and curricular gaps. **Settings:** Two academic and clinical sites in Vitória and Salvador, Brazil. **Attendees:** Fifty attendees (30 in Vitória, 20 in Salvador), comprising students, faculty, and health and social care professionals. **Methods:** The 30 h in-person course was delivered in late November (Vitória) and early December 2025 (Salvador) and addressed ‘wicked problems’ through value proposition development, design thinking, and prototyping. Facilitated by a Canadian faculty with English–Portuguese simultaneous translation. The course’s immediate evaluation used daily reflective exercises and a final participant survey. **Results:** Respondents’ reflections suggested a perceived reframing of innovation from a primarily technology-oriented concept toward a human-centered, context-responsive, and systems-oriented process. Respondents also identified areas requiring further curricular attention, including financial sustainability, implementation planning, and impact evaluation. **Conclusions:** Findings from this immediate post-course evaluation suggest that short-term, interprofessional innovation education may support perceived shifts in how participants understand health innovation. However, because the evaluation relied on self-reported, post-course qualitative data without pre/post or longitudinal measures, findings should be interpreted as perceived learning and exploratory curricular insight rather than demonstrated competency development or sustained implementation capacity.

## 1. Introduction

Innovation in healthcare is a fundamental driver of improved health outcomes and system responsiveness, particularly in complex public health systems where structural constraints, workforce capacity, and organizational culture interact to shape the implementation of new practices and technologies. As global health challenges grow in complexity, health and social care professionals must adopt evidence-informed methods and creative problem-solving to implement the most effective solutions. Integrating innovative competencies into health and social care professional education is essential for building resilient systems [1]. In Brazil, the Unified Health System (Sistema Único de Saúde [SUS]) provides a critical landscape for these advancements, yet its implementation is often hindered by regional disparities and resource constraints [2].

The SUS, as a universal and decentralized system, represents one of the world’s most ambitious public health frameworks [3], yet it continues to face significant operational challenges rooted in deep-seated regional inequalities [4]. Research indicates that the concentration of technological resources and specialized labor in Brazil’s South and Southeast creates a fragmented healthcare landscape, often leaving the North and Northeast regions with insufficient infrastructure to sustain high-level health innovations [5]. These structural disparities were acutely exposed during the COVID-19 pandemic, when chronic underfunding and limited local technical capacity severely tested the system’s resilience. Such regional asymmetries continue to be compounded by persistent resource constraints, hindering the SUS’s ability to effectively implement and scale evidence-based innovations across Brazil’s diverse geographic landscape [3,6]. Consequently, building innovation competencies in this context requires a focus on local resilience and the adaptation of solutions to overcome persistent systemic constraints, especially through the development of practitioner-led innovation training capable of responding to institutional, managerial, and socio-territorial disparities within the Brazilian health system.

To address these challenges, there is a growing interest in embedding innovation into Brazilian health and social care curricula. Research highlights that fostering a culture of innovation through interprofessional training and active learning is key to developing the critical thinking skills required for clinical leadership [7,8]. Key initiatives, such as the Fifth Strategic Plan for the Development of Epidemiology in Brazil (2025–2029) by the Brazilian Association of Collective Health [9], emphasize that these innovations must be socially referenced and committed to reducing inequities through collaborative, evidence-based interventions [10].

In alignment with this agenda, sponsored by Global Affairs Canada’s The Faculty Mobility for Partnership Building Program grant, faculty from an urban Canadian academic institution partnered with two Brazilian academic institutions to deliver a course aimed at strengthening health innovation. This collaboration was grounded in shared commitments to interprofessional learning and the development of sustainable innovation ecosystems. This manuscript reports on the delivery and immediate evaluation outcomes of the course, Evidence-based Innovation in Health, implemented in the Brazilian coastal cities of Salvador and Vitória. Accordingly, this paper presents a qualitative, pedagogically focused program evaluation of a short-term binational course in evidence-based health innovation, examining how participants perceived the relevance, applicability, and limitations of the educational model within the context of Brazil’s Unified Health System.

This paper is positioned as a qualitative, pedagogically focused program evaluation of a binational educational innovation course, rather than as a controlled assessment of competency acquisition or long-term implementation outcomes. Using immediate post-course evaluation data, the paper examines how attendees perceived the relevance, applicability, and limitations of evidence-based innovation education within the Brazilian health system context. It offers exploratory pedagogical insights into how short-term, interprofessional innovation training may support broader understandings of innovation and identify areas for future curriculum development.

### 1.1. Theoretical Frameworks

The design and delivery of the Evidence-based Innovation in Health course were guided by two key theoretical frameworks: Diffusion of Innovation and Experiential Learning Theory. These frameworks informed the pedagogical strategies, implementation approach, and evaluation of attendees’ engagement with innovation. Collectively, they provided a conceptual foundation for supporting attendees in adopting new ways of thinking, applying theory to practice, and disseminating innovation within their professional communities.

#### 1.1.1. Diffusion of Innovation Theory

Rogers’ Diffusion of Innovation (DOI) theory [11] offers a robust lens to understand how new ideas, practices, and technologies spread within complex social systems. The course aimed to introduce innovation concepts and create conditions that might support their later adoption within attendees’ academic and professional settings. DOI emphasizes five core characteristics that determine the rate of adoption: relative advantage, compatibility, complexity, trialability, and observability [11].

These attributes were intentionally embedded throughout the course structure to facilitate the uptake of innovation methodologies in the Brazilian context. For example, design thinking and value proposition generation activities highlighted the relative advantage of user-centered approaches in addressing persistent health challenges compared to traditional top-down models. The integration of practical, contextually relevant examples was intended to enhance compatibility with attendees’ experiences within the Brazilian SUS. Hands-on ideation and prototyping exercises supported trialability, enabling attendees to experiment with innovation processes in a low-risk environment before clinical application. Finally, pitch presentations enhanced observability by allowing attendees to articulate their proposed innovations, demonstrate how they had applied course concepts, and engage in peer and faculty dialog.

#### 1.1.2. Experiential Learning Theory

Kolb’s Experiential Learning Theory (ELT) informed the course structure, emphasizing that learning is a continuous process where knowledge is created through the transformation of experience [12]. The curriculum was designed to guide attendees through the four stages of the learning cycle: concrete experience, reflective observation, abstract conceptualization, and active experimentation [12]. Each component of the course was intentionally designed to support movement through this cycle. Workshops began with concrete experiences, such as identifying local wicked problems or participating in empathy mapping exercises, allowing attendees to anchor their learning in real-world scenarios relevant to their communities. Daily process evaluations served as structured opportunities for reflective observation, encouraging attendees to identify challenges, key insights, and areas for improvement. These reflections deepened their understanding of how innovation concepts applied to their evolving project work.

Classroom discussions and faculty-led mini-lectures facilitated abstract conceptualization, helping attendees connect their reflections to broader theoretical models, including design thinking, value proposition design, and other relevant entrepreneurial frameworks [13,14,15,16]. Attendees then engaged in active experimentation through prototyping, customer discovery simulations, and pitch development. This cycle repeated daily and culminated in the final pitch presentations, where teams synthesized their learning into actionable innovation proposals. By embedding ELT into the pedagogy, the course promoted deeper learning, skill integration, and the development of adaptive expertise, qualities essential for driving innovation in complex health systems.

Together, DOI theory and ELT provided a framework for understanding how attendees engaged with innovation concepts and for helping faculty adapt innovation teaching to local institutional and health-system contexts.

## 2. Materials and Methods

### 2.1. Course Description

The course Evidence-based Innovation in Health was delivered in two Brazilian cities: Salvador (state of Bahia; Federal University of Bahia [UFBA]) and Vitória (state of Espírito Santo; Federal University of Espírito Santo [UFES]). Each site received approximately 30 h of instruction led by a collaborative, interdisciplinary team. A professor in nursing delivered the in-person components alongside a physically present instructional team, while a professor in chemistry, who is the founding director of the Science Discovery Zone at Toronto Metropolitan University (Canada), facilitated virtually. This dual-delivery approach ensured cross-disciplinary perspectives on innovation, entrepreneurship, and problem-solving within health systems. To ensure language accessibility, two translators provided simultaneous interpretation in each city, and all teaching materials, including slide decks and video lectures (delivered in English with Portuguese transcripts and subtitles), were translated into Portuguese prior to delivery. Additionally, Brazilian faculty partners at each institution played pivotal roles in contextualizing the content, co-facilitating discussions, and supporting attendees’ engagement.

In both locations, advertisements were made in advance of the course offering, and all participants self-selected. In Vitória, 30 attendees successfully completed the course. The Salvador cohort began with 25 enrollees, of whom 20 completed the workshop, as five attendees discontinued their attendance due to scheduling conflicts they developed throughout the week. Attendees represented diverse academic backgrounds, including nursing, medicine, public health, and allied health fields, contributing to a deeply interprofessional learning environment. They also represented all levels of education, from undergraduate to graduate students and post-doctoral fellows, as well as genders. Prior to the in-person sessions, attendees completed preparatory readings and reflective pre-work intended to orient them to foundational concepts in innovation and evidence-informed decision-making.

At the start of the sessions, attendees were divided into groups of 4 to 6 people, ensuring that no professional background was represented within the group to allow for interprofessional collaboration and conversations. Through six-hour sessions together, delivered every day over a one-week period, the course introduced attendees to the nature of wicked problems in health systems and engaged them in identifying root causes and contextual factors contributing to these challenges. Attendees practiced ideation techniques to generate creative responses and learned how to articulate compelling value propositions. Sessions on design thinking emphasized empathy-driven problem framing, iterative prototyping, and user engagement. Analytical tools such as the Political, Economic, Social, Technological, Legal, and Environmental (PESTLE) framework [17] and the Strengths, Weaknesses, Opportunities, and Threats (SWOT) analysis [18] enabled attendees to assess broader sociopolitical and environmental factors that shape innovation in health settings. However, introducing multiple tools allowed attendees to encounter several complementary methods, but the compressed format limited opportunities to achieve proficiency or carry projects through implementation and evaluation. Throughout the course, attendees worked in small teams to develop innovation briefs, refine concepts through testing, and ultimately prepare pitch presentations delivered to their classmates on the final day on their chosen topic.

These methods were selected because the course focused primarily on early-stage innovation processes, including problem identification, contextual analysis, stakeholder engagement, ideation, and preliminary testing across professional disciplines. The pedagogical intent was not to solve a problem but rather to learn a methodology that can address wicked problems. Problem-Based Learning was not viewed as a direct alternative to these methods, but rather as a broader pedagogical approach that can incorporate similar collaborative and problem-centered activities. Quality-improvement frameworks [19] were also considered relevant; however, they are generally most applicable once a specific clinical or organizational process, and implementation target have been identified. Our methodology emphasizes problem discovery and early concept development, and therefore, formal quality-improvement methods were not included as a central component. Such frameworks may be more appropriately incorporated into a subsequent implementation-focused course or follow-up workshops.

### 2.2. Collaborative Delivery and Capacity-Building

A defining feature of the initiative was its intentional focus on long-term sustainability. Brazilian faculty members were integrated as co-facilitators and observers throughout the course delivery, with opportunities to participate in instructional planning, live facilitation, and debrief sessions. These activities were complemented by dedicated consultation periods in which Canadian educators supported colleagues in refining elective course proposals, adapting teaching materials, and exploring strategies to embed innovation principles into existing curricula.

### 2.3. Pitch Presentation Topics

Across the two sites, attendees engaged deeply with the prompt “How can healthcare be improved in Brazil?”, producing eight innovation proposals that reflected both structural system-level challenges and the lived experiences of Brazilian communities. The first project focused on strengthening transparency and patient autonomy within Brazilian SUS through a publicly governed digital platform that enables users to access real-time information on appointments, diagnostic tests, and surgical waitlists. The innovation framed ‘lack of transparency’ as a key driver of inequity and mistrust and positioned digital infrastructure to reduce wait times, mitigate corruption in queue management, and enhance citizen engagement with public healthcare, while adhering to national data protection legislation.

The second project emphasized the need to rebalance healthcare delivery toward prevention and health promotion, particularly for populations experiencing social vulnerability. This proposal introduced a mobile, community-based service model designed to deliver interprofessional health promotion activities directly within communities. By prioritizing primary healthcare (PHC), prevention, and intersectoral collaboration, the innovation sought to address chronic disease management, social isolation, and health inequities while reducing avoidable reliance on hospital-based care and higher-cost services.

The third project approached healthcare improvement through educational innovation, positioning academic institutions as key drivers of health system transformation. The proposed initiative focused on developing interprofessional appreciation of evidence-based innovation, leadership, and collaborative practice through structured, active-learning curricula. By integrating international perspectives, patient as a partner engagement, and applied innovation methodologies, the project framed education as a foundational strategy for building sustainable innovation ecosystems capable of generating locally relevant solutions to complex health system challenges.

The fourth innovation addressed the growing burden of chronic non-communicable diseases by proposing a digital platform to support health education, self-management, and continuity of care within PHC settings. The platform emphasized health literacy, patient engagement, and personalized support through features such as medication reminders, lifestyle guidance, progress tracking, and evidence-based educational content. By aligning adult learning principles with culturally adapted communication, the proposal positioned digital education as a scalable strategy to improve treatment adherence, promote the empowerment of individuals to engage in self-care, and reduce preventable demand on emergency and specialized services.

The fifth project focused on improving access to PHC for working adults who face structural barriers due to inflexible clinic hours. The proposed innovation involved extending PHC service hours into the evening to accommodate individuals unable to attend appointments during standard working hours. By leveraging existing infrastructure, integrating supervised students and patients into care delivery, and aligning funding with national access-expansion policies, the model aimed to improve first-contact access, strengthen preventive care, reduce absenteeism, and alleviate pressure on urgent and emergency care services.

The sixth project introduced a strategic digital solution to mitigate critical fragmentation and communication deficits within the SUS. This was achieved by proposing the development of an online platform engineered to integrate and leverage existing data infrastructure rather than creating new systems. The platform is structured with three specialized interfaces: the User Interface enhances patient autonomy by providing access to personal health records and appointment management; the Healthcare Professional Interface supports informed clinical decisions with summarized patient histories and clinical alerts; and the Healthcare Manager Interface provides actionable intelligence by systematizing dispersed data, integrating both primary indicators and automated linkages with official databases.

The seventh project addressed the significant operational challenge of prolonged waiting periods for medium- and high-complexity diagnostic exams, such as Computed Tomography, Magnetic Resonance Imaging, and specialized ultrasonography, within the Brazilian SUS. The project’s innovation is the standardization of maximum allowable timeframes for scheduling and executing these complex exams, defined by both the exam type and clinical priority, with the core methodology involving the creation of an indicator for continuous service monitoring to ensure accountability and efficiency in achieving faster diagnoses and a fairer queue.

The eighth project was a health education initiative centered on promoting early-life health interventions to ensure healthy aging. It is designed to address the public health concern of unhealthy and rapid aging that is perpetuated by a critical lack of lifestyle-focused health education. The target audience comprises public school teenagers who need to form new habits to secure healthy longevity. The core solution is a multidisciplinary health education project that mobilizes adolescents to participate in health practices through the co-creation of a technological product, leveraging their innate creativity and technological literacy.

Collectively, these eight innovation proposals illustrate the range of health system challenges attendees identified as priorities in the Brazilian context. The projects addressed issues related to digital integration and transparency (Projects 1, 4, and 6), access to care, health promotion (Projects 2, 4, and 8), chronic disease management, diagnostic wait times (Projects 5 and 7), and interprofessional education (Project 3). Although preliminary and conceptual in nature, the proposals suggest that attendees were able to apply course concepts to locally relevant problems and to frame innovation as encompassing technological, social, organizational, and educational forms of change.

### 2.4. Evaluation Methods and Collection

This study was undertaken as an educational program evaluation intended to assess the perceived relevance, applicability, and areas for improvement of a binational course delivered at two Brazilian sites. Qualitative description and conventional qualitative content analysis were used as the methodological and analytic approaches for examining the open-ended evaluation responses. Thus, program evaluation describes the purpose of the inquiry, whereas qualitative description describes how the evaluation data were analyzed. The Toronto Metropolitan University Research Ethics Board was consulted and determined that a formal research ethics review was not required because the activity was undertaken as a pedagogical program evaluation.

The course evaluation included daily process-oriented reflections and a final outcome-oriented survey (see Supplementary Materials). The evaluation questions were developed for local pedagogical feedback and course-improvement purposes. They were not intended as psychometric instruments, competency assessments, or transferable evaluation tools, and no claims regarding validity, reliability, or cross-cultural adaptation are made.

Each day concluded with a reflective process evaluation intended to capture attendees’ immediate experiences and evolving learning needs. Attendees were asked three open-ended questions: (1) What are you struggling with when it comes to the content we learned and applied today? (2) What aspect of the course would you like to see improved for the next day? and (3) What is one key piece of learning that stood out to you most today? These reflections provided real-time insights into respondents’ cognitive and emotional engagement and informed minor adjustments to daily facilitation: These responses were not part of the analysis below.

Across both sites, 55 individuals initially enrolled or began participation in the course. Thirty attendees completed the Vitória offering, and 20 completed the Salvador offering, for a total of 50 course completers. All 50 course completers were invited to complete the final evaluation on the last day of the course. Participation in the evaluation was voluntary and was not required for course completion or associated with any academic or professional consequences. Responses were de-identified and were submitted electronically. Twenty-five attendees submitted responses. The final analytic sample therefore included 24 respondents, representing 48% of course completers.

On the final day, attendees completed a more comprehensive outcomes evaluation survey designed to assess perceived impact and knowledge acquisition of the intervention. This survey included the following questions: (1) Is there something you hoped to learn that was not covered this week, or a topic that was covered but on which you would have liked more time? (2) If there was one change you could make to the course, what would it be? (3) What was the most impactful lesson or idea that you took away from this workshop? (4) How will you apply the insights and skills you gained during this week to your personal and professional goals? (5) What advice would you give to someone interested in pursuing social innovation work based on your experience in this workshop?

These evaluations captured respondents’ evolving perspectives on innovation, their appreciation for the applied learning model, and their recognition of the value of interprofessional collaboration.

#### Evaluation Analysis

The outcome evaluation data were analyzed using a qualitative descriptive approach to provide a comprehensive account of respondents’ perceptions of the course, the relevance of its components, and the anticipated application of innovation skills in their professional contexts. Given that the majority of attendees submitted their responses in Portuguese (13), while a smaller number wrote in English (11), the analytic process required an additional step to ensure that all information could be examined in a consistent manner. Portuguese-language responses were translated into English to facilitate analysis within a common language dataset. Translation was undertaken collaboratively by bilingual members of the instructional team and Brazilian faculty partners who were familiar with the course context. The purpose of translation was to preserve participants’ intended meaning for qualitative analysis rather than to create or validate a cross-culturally adapted research instrument.

Following translation, the full corpus of responses from both sites was compiled and analyzed using conventional qualitative content analysis [20]. Conventional content analysis was selected because little was known about participants’ experiences with this educational program in the Brazilian context, allowing categories and themes to emerge inductively from the data rather than being guided by a pre-existing framework. NVivo 15 was used to organize the translated responses, support line-by-line coding, manage emerging codes, and facilitate comparison of coded segments across the dataset.

Two members of the instructional team independently read all translated responses several times to achieve immersion in the data and develop initial impressions. The unit of analysis consisted of individual meaning units, ranging from words and phrases to complete sentences that conveyed a single idea relevant to participants’ learning experiences. The coders then conducted line-by-line open coding in NVivo. Codes were generated inductively from the data, using participants’ language where appropriate while also applying interpretive labels when needed to capture underlying meaning. New codes were created throughout the analysis as additional concepts emerged.

Regular analytic meetings were held to compare coding decisions, discuss discrepancies, and iteratively refine the coding framework. During these meetings, code definitions were clarified, overlapping codes were merged, conceptually distinct codes were separated, and the evolving codebook was updated to improve consistency in coding across the dataset. When disagreements arose, the coders revisited the original responses and reached consensus through discussion rather than relying on quantitative measures of intercoder agreement. If consensus could not be reached through a discussion, a third researcher was invited to share their perspective. NVivo was used to support this process by organizing codes, coded excerpts, and category development; however, all analytic decisions were made by the research team through discussion and consensus.

Trustworthiness was supported through independent initial coding by two coders, iterative comparison and consensus discussion, attention to divergent and less-common responses, consultation with bilingual Brazilian collaborators, and re-examination of original Portuguese responses when meaning was uncertain. Because this was a bounded, one-time program-evaluation dataset, recruitment was not continued to achieve data saturation. Participant checking was not undertaken because responses were collected through a de-identified, one-time evaluation process.

Following completion of coding, related codes were compared for conceptual similarity and grouped into broader descriptive categories. Categories were continuously reviewed against the original coded data to ensure they accurately represented participants’ accounts while remaining internally coherent and distinct from one another. Higher-order themes were then developed by examining patterns across categories and identifying overarching concepts that captured participants’ shared experiences and perspectives. Through this iterative process, themes reflecting participants’ perspectives on innovation learning, professional empowerment, and the perceived applicability of innovation methodologies in real-world health system contexts were developed.

The final themes synthesized attendees’ reflections on the value of experiential and collaborative learning, their growing confidence in applying innovation methodologies, and their intentions to integrate course concepts into academic, clinical, or community-based contexts. The themes also illuminated areas where attendees perceived the need for expanded content coverage or extended instructional time. This set of evidence informed the interpretation and discussion presented in this manuscript and contributed to recommendations for sustaining and scaling innovation education within the partner institutions.

The analysis was conducted by K.M. and R.R., both of whom were members of the instructional team and were involved in delivering, facilitating, and translating the course. Participants therefore knew them in their educational roles and understood that the evaluation would inform course improvement. Neither coder had an established relationship with participants before the initiative/describe any prior relationships. Their nursing, educational, and Brazilian contextual perspectives informed the interpretation of the data. This insider positioning provided a detailed understanding of the educational and linguistic context but also created a risk that favorable interpretations of the course would be privileged. The team sought to mitigate this risk through independent initial coding, consensus discussions, attention to critical and divergent responses, and consultation of the original Portuguese-language responses when meaning was uncertain. Nevertheless, researcher involvement in the intervention remains a potential source of interpretive and confirmation bias. Reporting of the qualitative component was reviewed against the applicable COREQ criteria. Items specific to interviews and focus groups were not applicable to this written, open-ended program evaluation.

## 3. Results

### Evaluation Evidence

**Figure 1:**
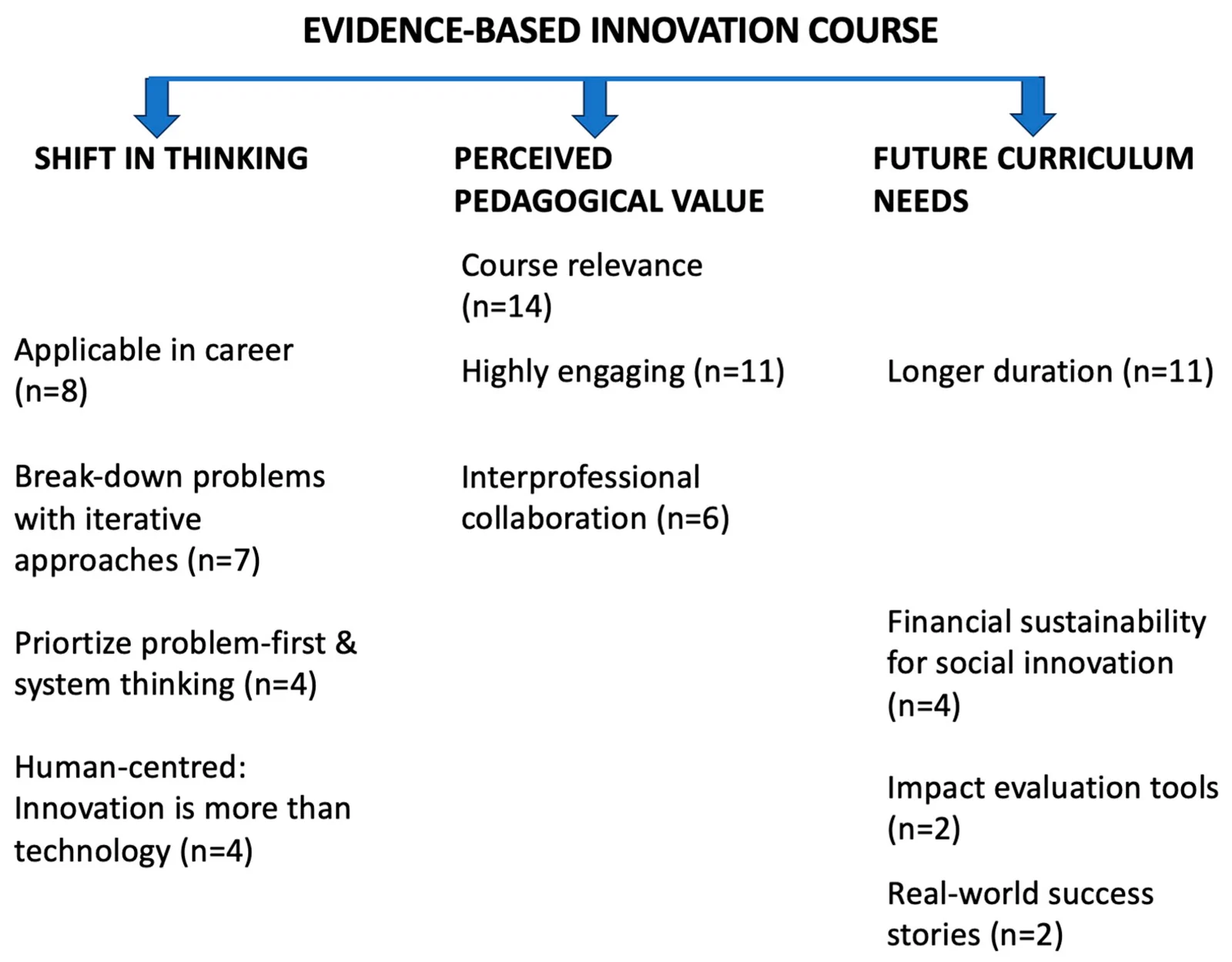
Summary of participant perceptions and recommendations from the immediate post-course evaluation (n = 24). Respondents could contribute to more than one category, and counts indicate the number of respondents whose answers were coded to each category, not mutually exclusive prevalence estimates.

This analysis focuses on understanding how participation in the course may have influenced attendees’ conceptualization of innovation, perceived learning, and readiness to apply innovation approaches within their professional contexts. The findings are organized around three key areas: (1) shifts in how innovation is understood, (2) perceived strengths of the educational model, and (3) limitations and implications for future implementation. To improve transparency and readability, the most frequently reported themes from the post-course evaluation are summarized in Figure 1. This visual summary complements the narrative presentation of findings below and highlights the relative prominence of the participant feedback.

The respondent group was predominantly female and comprised healthcare professionals and researchers at the postgraduate level, including several doctoral candidates. Reflecting the interprofessional focus of the workshop, the respondents represented a broad range of clinical and administrative backgrounds: nursing, complemented by health and social care providers in nutrition, physiotherapy, medicine, dentistry, physical education, and biomedicine, as well as professionals in health administration. While this diverse composition ensured that the feedback encompassed a wide variety of perspectives, detailed demographic characteristics were not collected as research variables because the form was designed for local course evaluation. Consequently, only aggregate characteristics available from course records are reported.

Outcome evaluations indicated that respondents viewed the course as relevant and useful to their professional trajectories. Their responses reflected a conceptualization of innovation that extended beyond technology-centered approaches to include systemic and human-centered processes grounded in problem identification, stakeholder engagement, and iterative solution development. While the majority of respondents (n = 14) stated that the course met or exceeded their expectations, a distinct subset of six attendees identified specific technical gaps they wished had been explored in greater depth, particularly regarding the financial sustainability of social projects and the quantitative measurement of results. As one respondent observed, “the financial part of the project is already an approach with a deficit in most Brazilian health professional graduations,” and it remained an area that needed more attention in the course. Additionally, two respondents expressed a desire for more concrete tools for evaluation, with one respondent noting a need for more time dedicated to measuring outcomes and long-term results: “I was hoping to learn more about practical impact evaluation in social innovation projects, especially how to measure outcomes and long-term results using clear indicators and data.” There were also suggestions from two respondents to include real-world examples of successful past projects to help respondents visualize practical pathways from design to implementation.

When asked to suggest changes to the course, the most frequent recommendation was to extend the total duration of the workshop to allow for a less hurried pace. Eleven respondents felt that while the content was rich, more time was necessary. As one respondent mentioned, “The content was rich and the methodology highly engaging but having more time would allow for deeper exploration of each stage and a more complete experience with prototyping and refinement of solutions.” Beyond time allocation, structural suggestions included integrating the preparation of the final pitch into the daily lessons rather than leaving it for the final day. As one respondent recommended, “it would be beneficial to elaborate each part of the presentation concurrently with the classes.” Other suggestions focused on increasing collaborative opportunities. One respondent said, “If I could make one change to the course, I would include more hands-on, collaborative activities, such as group problem-solving, real-case simulations, and structured peer feedback, to better connect theory with practice.”

The respondents identified several transformative lessons, primarily focusing on the demystification of innovation and the adoption of structured problem-solving techniques. Four respondents commented that a central takeaway was the realization that overwhelming social challenges become manageable when broken down into smaller, actionable components. According to one respondent, the most impactful idea was that “complex problems can only be meaningfully addressed when we break them down into smaller components and take the time to understand the entire system”. Another participant similarly added, “Problems—even the biggest and most complex ones—can be minimized or even solved when we think in terms of focused strategies.” Another significant theme, highlighted by four respondents, was the shift toward human-centered design, where innovation is defined by empathy rather than just technology. As one respondent reflected, they learned that “social innovation is not just about creating something new but about solving real problems in a human-centered, ethical, and sustainable way”.

Regarding the application of their new skills, respondents planned to integrate these methodologies into both their academic research and their daily clinical or administrative roles. One respondent noted that the tools would be directly applied to their postgraduate studies: “I can use it to improve my doctoral project.” In professional settings, seven respondents focused on using structured analysis to improve organizational efficiency and patient care. One respondent mentioned that they would use these skills to organize and systematize the resolution of problems in their workplace. As an example, one participant shared, “I work as a home-care physiotherapist and I am implementing technological assessment with inertial sensors in my sessions. The tools I learned will help me organize this implementation for better delivery.” Others (n = 3) emphasized a newfound focus on the patient experience, intending to be more attentive to patients and their issues by including them more directly in the process of creating solutions.

In offering advice to others interested in health and social innovation, the respondents emphasized the importance of context, collaboration, and starting small. The most common advice was to prioritize understanding the problem over jumping to a solution. As one respondent advised, “My advice to anyone interested in pursuing social innovation work is to start with the problem, not the solution. Take time to understand the social context, listen to the people affected, collaborate with others, and remain committed to ethical, long-term impact rather than quick results.” Respondents (n = 6) also highlighted the interprofessional nature of the work, noting that health and social innovation is fundamentally a collective effort that requires networking and engagement with diverse stakeholders. This can include patients. Particularly, one participant outlined, “Always listen to the people who experience the problem in their daily lives, and think about how we can positively impact their reality together.” Lastly, they (n = 3) encouraged others to embrace the iterative process. As one participant shared, “Social innovation doesn’t consist of having the perfect idea from the beginning, but in taking advantage of solutions and adapting continuously”.

The final pitch presentations provided examples of how attendees applied course concepts to locally relevant health-system challenges. The pitches can be described as instructional outputs and are used illustratively. Although conceptual in nature, the pitches suggested that attendees were able to draw on user-centered design, value proposition development, and evaluative thinking to develop preliminary innovation proposals. Projects addressed issues such as health inequities, digital health literacy, access to mental health services, and community-based chronic disease management. As one respondent concluded, “innovation is only possible from a human perspective that is unsettled by social challenges.” Another participant added, “My advice would be: embrace uncertainty and trust the process. Social innovation is not linear, and meaningful ideas often emerge only after exploring many angles of a problem, listening deeply to stakeholders, and being willing to revise initial assumptions.”

## 4. Discussion

This qualitative, pedagogically focused program evaluation offers insight into how attendees experienced a short-term, binational course in Evidence-based Health Innovation within the Brazilian health-system context. Rather than serving as a measure of long-term competency development or implementation impact, the findings provide a snapshot of attendees’ immediate perceptions of the course, including how they understood innovation, what aspects of the learning model they found valuable, and what additional supports they felt would be needed to move from methodological training toward idea implementation. Across the responses, attendees emphasized the value of contextualized, interprofessional, and experiential learning, while also identifying the need for greater attention to financial sustainability, impact evaluation, and implementation planning. Given the introductory and intensive nature of the course, these areas may be most appropriately addressed through staged follow-up programming rather than compressed into the initial methodological workshop [16].

### 4.1. Interpretation Through the Theoretical Frameworks

The findings can be interpreted in relation to Kolb’s Experiential Learning Theory, although the present evaluation did not directly measure progression through the stages of the learning cycle. The course design provided concrete experiences through the identification of locally relevant wicked problems, empathy-mapping activities, and engagement with health system challenges within the Brazilian SUS. Opportunities for reflective observation were incorporated through team discussions and daily reflective exercises, while faculty-led instruction and the application of design-thinking and value-proposition concepts supported abstract conceptualization. Prototyping, project refinement, and final pitch presentations provided opportunities for active experimentation. Respondents’ descriptions of breaking complex problems into smaller components, beginning with the problem rather than the solution, and applying structured approaches in their professional settings are consistent with this experiential sequence. At the same time, respondents’ requests for a longer and less hurried course suggest that the condensed format may have limited the time available for repeated experimentation, feedback, and refinement. The findings therefore support the relevance of experiential learning for introductory innovation education while also suggesting that fuller engagement with the learning cycle may require additional time or staged follow-up programming.

Rogers’ Diffusion of Innovation (DOI) Theory also provides a useful lens for interpreting the course design and respondents’ perceptions. The use of challenges grounded in the SUS and the involvement of Brazilian faculty were intended to enhance the compatibility of the innovation methods with attendees’ professional and institutional contexts. Small-scale ideation and prototyping activities supported trialability by allowing attendees to test approaches in a low-risk educational setting, while the final pitch presentations increased observability by making proposed applications visible to peers and faculty. Respondents’ descriptions of human-centered, problem-first, and collaborative approaches as useful to their professional trajectories may reflect the perceived relative advantage of these methods over narrowly technology-centered or solution-first approaches. Conversely, requests for more time and greater attention to implementation, financial sustainability, and evaluation indicate that the number and complexity of the methods introduced may have constrained more advanced application within the short-course format.

These findings should not, however, be interpreted as evidence that diffusion or adoption occurred. DOI informed the educational design and provides a framework for interpreting attendees’ immediate perceptions, but the evaluation did not assess subsequent uptake, dissemination within professional communities, or participants’ roles as early adopters. Longitudinal follow-up would be required to determine whether the methods introduced during the course were later adopted, adapted, or shared within academic, clinical, or organizational settings.

### 4.2. Interpretations from Participant Feedback

The feedback received from respondents in their evaluation of the Evidence-based Innovation in Health course highlights the value of international and interprofessional collaboration in advancing health innovation education. Recent research suggests that these strategic alliances are essential for fostering innovative perspectives within global health, particularly in the context of sustainable development goals [21]. By combining evidence-informed pedagogical methods with principles of entrepreneurship and design, the course provided Brazilian students and faculty with tools to navigate and influence complex health systems. The strong engagement observed across both sites suggests the demand for innovation training within health and social care professional education in Brazil.

A notable observation in the evaluation was that innovation was often discussed in relation to technology, digital tools, or product development. This may reflect broader health-system discourse, in which innovation is frequently equated with technological modernization. However, the course evaluation suggests that attendees also began to recognize innovation as a broader process that can include social, organizational, educational, and practice-based change. This broader framing is important because many challenges in health care are not solved by technology alone; they require attention to workflows, relationships, communication, implementation conditions, and the lived realities of patients, communities, and frontline professionals. This attitudinal shift is in line with current scholarship that emphasizes that in public systems, the most sustainable interventions are those rooted in empathy and iterative, collaborative co-design. This aligns with the findings of Suarez-Herrera et al., who argue that in resource-constrained environments like the Brazilian SUS, social innovation must prioritize human-centered, ethical, and sustainable solutions over technological complexity [22].

By framing innovation as a manageable and iterative process, the workshop may have helped respondents see complex health-system challenges as more approachable within resource-constrained settings such as the SUS, as suggested by Harris et al.’s argument that frugal innovation competencies are essential for the promotion of empowerment in health workers to solve systemic inefficiencies without waiting for large-scale external funding [23].

The respondents’ appreciation for interprofessional dialog reinforces the necessity of cognitive diversity when addressing “wicked problems” inherent in the health sector. The finding that working with peers from diverse backgrounds enhanced the ability to view challenges from multiple perspectives is supported by recent studies on collective intelligence in health education, which argue that cultivating such collaborative intelligence is essential for sustainable healthcare [24,25]. This multidisciplinary approach, composed here of nurses, doctors, physiotherapists, and administrators, mirrors the collaborative practice models recommended by international health bodies to drive equitable, systemic change.

Respondents’ comments also pointed to a potential curricular gap related to financial sustainability and impact measurement. Several respondents noted that questions of funding, metrics, and long-term feasibility were difficult to address within the scope of the introductory workshop, and some described these areas as broader gaps in health professional education. This aligns with literature suggesting that managerial and financial competencies are often underdeveloped in health training contexts. Rather than suggesting that all of these topics should be compressed into an introductory innovation course, the findings point toward the value of staged or follow-up programming focused on implementation planning, sustainability, evaluation, and partnership development. Future course iterations should include literature or training on quantitative success indicators/metrics and collaboration with local incubators to include guest speakers who have been successful with funding. In addition, a subsequent implementation-focused course could incorporate quality-improvement frameworks once participants have identified a defined process, measurable target, and feasible intervention.

The tension identified in the evidence between the “intensive bootcamp” format and the respondents’ desire for a “less hurried pace” highlights a universal challenge in professional development: the trade-off between immersion and retention. This course was intended to expose attendees to a novel methodology for evidence-based innovation, with the intent to be fast-paced, such that it could be delivered in a weeklong workshop. While the short duration was sufficient to inspire a mindset shift, the respondents’ request for continuous, collaborative engagement frameworks suggests that deep competency requires ongoing support. This observation is corroborated by Tieosapjaroen et al., who conducted a global systematic review of health designathons and innovation events [21]. Their research identifies time constraints as a primary barrier to high-quality project outcomes, noting that condensed timelines often limit the ability of attendees to move beyond ideation into the critical phases of prototyping and refinement. The authors emphasize that for innovation training to bridge the gap between classroom concepts and clinical reality, programs must incorporate sustained mentorship and follow-up structures.

The respondents’ strong advice to “start with the problem” and to “understand the social context” before proposing solutions demonstrated an intuitive grasp of the contextual determinants of health. Moullin et al. emphasize that in implementation science, the fit between an innovation and the local context (including cultural values and resource availability) is the single strongest predictor of success [26]. The evidence shows that this course’s evidence-based methodology fostered a commitment to contextual responsiveness, ensuring solutions are tailored to local realities. This is particularly crucial for the SUS, where imported solutions often fail.

This initiative further highlights the importance of contextual relevance. Brazilian faculty played a crucial role in grounding the content in local health system realities, while the integration of translation allowed for deeper conceptual understanding and more inclusive discourse. The feedback provided by respondents suggests that innovation education is most effective when it is experiential, interactive, and explicitly connected to local challenges.

In addition to teaching and consultation activities, project leads met with institutional authorities at UFBA and UFES to discuss ongoing collaboration. These meetings included planning for the development of Memoranda of Understanding, which are expected to formalize future student exchanges, faculty mobility, and joint research initiatives. These discussions further reinforced the shared commitment to building a sustainable, internationally connected innovation ecosystem grounded in evidence-based pedagogy.

The capacity-building components of this initiative were an important part of its design. Brazilian faculty participation, consultation periods, and discussions about future collaboration may support longer-term integration of innovation education within partner institutions. However, these potential institutional outcomes were not evaluated in the present study and should be understood as future directions rather than demonstrated impacts [27]. The parallel discussions regarding Memorandums of Understanding and future collaborations indicate strong institutional alignment and a shared vision for ongoing partnership.

Taken together, these findings suggest that the value of this initiative lies not only in what was taught but in how innovation itself was reframed. By shifting the focus from technology to context, from solutions to problem definition, and from individual ideas to system-level thinking, the course model may offer a useful approach for introducing innovation capacity-building in similar educational contexts. These findings support the view that innovation can be framed not only as a specialized technical skillset but also as a broader competency relevant to health professionals working within complex and inequitable systems.

The potential transferability of this educational model lies primarily in its pedagogical design rather than in the direct replication of the course content or findings. Features that may be adaptable to other health-professions education settings include the involvement of local faculty as co-facilitators, attention to linguistic accessibility, interprofessional teamwork and composition, experiential work focused on locally identified health-system problems, and the use of staged learning that distinguishes foundational innovation methods from later implementation-oriented training. However, the relevance and application of these features depend on local adaptation and scoping in advance of course delivery. The present course was shaped by the governance, financing, and regional inequalities of the Brazilian Unified Health System, as well as by the institutional relationships between the participating universities, the professional composition of the participants, and the short-term mobility-grant structure. Consequently, the findings should not be assumed to apply directly to other health systems or educational settings, although the underlying design principles may provide a useful starting point for contextually adapted innovation education when tackling wicked problems. Furthermore, the contribution of this evaluation is not evidence that a single course produces enduring innovation competence. Rather, it identifies three potentially useful design considerations for international health-innovation education: innovation content should be co-contextualized with local partners; short experiential and interprofessional activities may help broaden innovation beyond technological solutionism; and foundational innovation training should be linked to later instruction in implementation, finance, and evaluation. These insights may inform educators designing similar introductory initiatives in complex public health systems, while requiring adaptation to local institutional and cultural conditions.

### 4.3. Limitations

This study presents several limitations that should be acknowledged. First, the evaluation relied on self-reported participant reflections and post-course survey responses, which capture immediate perceptions of learning, relevance, and applicability rather than objective measures of competency development, behavioral change, or long-term implementation outcomes. Because the evaluation was conducted immediately following the course, the findings should be interpreted as perceived learning and perceived readiness rather than evidence of sustained professional or institutional impact. Findings represent immediate post-course perceptions and reflections and should not be interpreted as evidence of sustained educational outcomes, behavioral change, or long-term professional practice impacts. In addition, the context-specific nature of the intervention, including its grounding in the Brazilian SUS and the structure of the binational academic partnership, limits the extent to which the findings can be transferred directly to other educational or health-system settings.

Second, the evaluation did not include pre-/post-measures, validated competency instruments, or longitudinal follow-up. As a result, it was not possible to determine whether participants’ understandings of innovation changed over time, whether reported insights translated into practice, or whether the innovation proposals developed during the course were eventually implemented within the Brazilian health system. Future research should therefore incorporate longitudinal and mixed-method evaluation designs, including pre-/post-assessment, follow-up interviews, and tracking of project development beyond the course.

Third, as previously mentioned, detailed demographic characteristics were not collected as research variables because the form was designed for local course evaluation. However, limited participant characteristics were collected and reported on in the manuscript, such as profession type. Due to this, we were able to achieve a respondent group that was heterogeneous, including students, faculty, and health and social care professionals from different disciplinary and institutional contexts. While this diversity was appropriate for an interprofessional educational initiative, the de-identified nature of the evaluation responses prevented analysis of discipline-specific or role-specific patterns. It was therefore not possible to determine whether the course was experienced differently by students, faculty, clinicians, administrators, or other professional groups.

Finally, several potential sources of bias should be considered. The modest response proportion may have introduced non-response bias, as attendees with particularly positive or negative experiences may have been more likely to complete the evaluation than those with more neutral perspectives. Consequently, the findings may overrepresent stronger opinions regarding the course while underrepresenting the experiences of less-engaged participants or those who chose not to complete the evaluation. Because responses were collected anonymously as part of a routine program evaluation, demographic or participation characteristics of non-respondents were unavailable, preventing comparison between respondents and non-respondents. As a result, the representativeness of the findings cannot be determined, and the results should be interpreted as reflecting the perspectives of participants who elected to provide feedback rather than all course attendees. In line with this, it is vital to mention that social desirability and enthusiasm bias may also have influenced responses, particularly because the evaluation was completed shortly after the course. In addition, members of the instructional team were involved in translation and analysis, which may have introduced interpretive or confirmation bias despite the use of collaborative review, consensus discussions, and consultation with bilingual collaborators. Translation from Portuguese to English may also have resulted in some loss of nuance, although efforts were made to preserve contextual and conceptual meaning.

### 4.4. Implications for Education, Future Research, and Clinical Practice

Taken together, the findings reported here suggest several implications for future iterations of evidence-based innovation education. First, attendees’ responses reinforce the importance of grounding innovation training in local context, frontline realities, and the constraints of the health system in which innovation is expected to occur. Second, the course appeared to support attendees in reframing innovation as a human-centered, collaborative, and iterative process rather than primarily a technological or product-oriented activity. Third, respondents’ comments point to the need for future curricula to extend beyond problem framing and ideation by incorporating more explicit attention to implementation planning, financial sustainability, evaluation, and systems-level feasibility. These implications should be interpreted as exploratory and context-specific, given the immediate, self-reported nature of the evaluation data.

The evidence from this evaluation offers useful, though preliminary, implications for education, research, and practice. From an educational perspective, the findings suggest that health and social care curricula may benefit from more explicit integration of innovation-oriented learning, particularly when such learning is grounded in local health-system realities and delivered through experiential, interprofessional approaches. A key implication is the need to frame innovation broadly (from the onset), not only as technological development, but also as social, organizational, educational, and practice-based change. This distinction is especially relevant in health systems where meaningful improvement may depend as much on change management, implementation capacity, and stakeholder engagement. Future curricula could therefore position innovation as a practical set of competencies related to problem framing, evidence use, collaborative design, implementation planning, evaluation, and reflection. In this way, innovation education may help learners approach complex health-system challenges with greater attention to equity, context, the precise root of the problem, and feasibility.

For clinical and organizational practice, the findings suggest that innovation training may be most relevant when it reflects the realities of frontline care. Attendees’ responses emphasized the importance of understanding problems before proposing solutions, engaging those affected by the problem, and adapting ideas to the constraints and opportunities of the local setting. These insights align with participatory and human-centered approaches to change, in which health professionals, patients, communities, and organizational stakeholders are viewed as contributors to the design and refinement of new practices. Although this evaluation does not demonstrate changes in clinical behavior or implementation outcomes, it suggests that educational initiatives of this kind may help prepare participants to think more critically about how innovations could be developed and adapted in practice.

Finally, the findings point to several directions for future research. Because this evaluation captured immediate, self-reported perceptions, future studies should examine whether and how participation in innovation education influences longer-term professional practice, research development, interprofessional collaboration, or implementation activity. Future work could also incorporate pre-/post-measures, longitudinal follow-up, mixed-method designs, or case tracking of innovation projects as they move beyond the classroom. Respondents’ comments regarding financial sustainability, impact measurement, and implementation planning also suggest the need for further research into how health professionals develop the practical competencies required to move promising ideas toward feasible and sustainable change.

## 5. Conclusions

This qualitative, pedagogically focused evaluation suggests that a short-term, binational educational initiative can serve as a useful model for introducing health professionals, students, and faculty to evidence-based innovation in a contextually grounded and interprofessional manner. Participants’ reflections indicated that the course may have helped broaden understandings of innovation beyond technology or product development toward a more human-centered, collaborative, and systems-aware approach to change. Within the Brazilian health-system context, this broader framing is particularly important because meaningful innovation may involve social, organizational, educational, and practice-based improvements as much as technological solutions.

At the same time, the evaluation highlights opportunities for future curriculum development. Respondents’ interest in financial sustainability, implementation planning, evaluation, feasibility, and impact metrics suggests that learners may benefit from a staged approach to innovation education. Rather than attempting to cover all aspects of innovation within a single intensive introductory course, future programming could distinguish between foundational training in evidence-based innovation methods and follow-up learning focused on implementation, sustainability, and evaluation. A second-stage workshop, longitudinal mentorship model, or follow-up course delivered after participants have had time to refine their ideas could provide a more appropriate structure for supporting the transition from early-stage concept development to feasible and sustainable practice change.

Because the findings are based on immediate, self-reported post-course reflections, they should be interpreted as exploratory and context-specific rather than as evidence of demonstrated competency development or long-term impact. Future research should examine whether and how staged or follow-up models of innovation education influence professional practice, interprofessional collaboration, faculty capacity-building, and the development or implementation of innovation projects over time.

## Data Availability

The original contributions presented in this study are included in the article. Further inquiries can be directed to the corresponding author.

## Supplementary Materials

The following supporting information can be downloaded at: https://www.mdpi.com/article/10.3390/nursrep16080279/s1, Supplementary File: Final Outcome-Oriented Survey.

## Public Involvement Statement

No public involvement in any aspect of this research.

## Guidelines and Standards Statement

The qualitative component of this program evaluation was reviewed and reported with reference to the applicable items of the Consolidated Criteria for Reporting Qualitative Research (COREQ) and the Standards for Reporting Qualitative Research (SRQR). Because data were collected through written responses to open-ended evaluation questions rather than interviews or focus groups, COREQ items concerning interviewer characteristics, repeat interviews, audio or visual recording, field notes, interview duration, transcripts, and the presence of non-participants during data collection were not applicable.

## Abbreviations

The following abbreviations are used in this manuscript:

SUS	Sistema Único de Saúde
PHC	Primary Healthcare
UFES	Federal University of Espírito Santo
UFBA	Federal University of Bahia
DOI	Diffusion of Innovation
ELT	Experiential Learning Theory
PESTLE	Political, Economic, Social, Technological, Legal, and Environmental
SWOT	Strengths, Weaknesses, Opportunities, and Threats
