# Supplementary material for: Advancing Evidence-Based Health Innovation in Brazil: A Qualitative Program Evaluation of a Binational Educational Initiative to Build Interprofessional Capacity
Source: Nurs Rep. 2026 Aug 11;16(8):279. doi: 10.3390/nursrep16080279 (PMC13516740; doi:10.3390/nursrep16080279)
Supplement: Supplementary file 1 [file nursrep-16-00279-s001.zip › nursrep-4398656-supplementary.pdf]

Supplementary Materials: Final Outcome-Oriented Survey

1. Is there something you were hoping to learn that was not covered this week or a topic that was covered but would have like to spend more time on?

2. If there was one change you could make to the course, what would it be?

3. What was the most impactful lesson or idea that you took away from this workshop?

4. How will you apply the insights and skills you gained during this week to your personal and professional goals?

5. What advice would you give to someone who is interested in pursuing social innovation work based on your experience in this workshop?
